# Mycobacterium bovis Infection of a Deep Brain Stimulation System Following Intravesical Bacillus Calmette-Guérin (BCG) Instillation

**DOI:** 10.3233/JPD-230426

**Published:** 2024-07-23

**Authors:** Linda E. Spruijt, Arne Mosch, Carel F.E. Hoffmann, Cees van Nieuwkoop, Jasper D. Tijsterman, Rodi Zutt, Niels A. van der Gaag, M. Fiorella Contarino

**Affiliations:** a Department of Neurology, Haga Teaching Hospital, The Hague, The Netherlands; b Department of Neurosurgery, Haga Teaching Hospital, The Hague, The Netherlands; c Department of Internal Medicine, Haga Teaching Hospital, The Hague, The Netherlands; d Department of Urology, Haga Teaching Hospital, The Hague, The Netherlands; e University Neurosurgical Center Holland, The Netherlands; f Department of Neurology, Leiden University Medical Center, Leiden, The Netherlands

**Keywords:** Parkinson’s disease, deep brain stimulation, Bacillus Calmette Guerin, mycobacterium bovis, infection

## Abstract

Deep brain stimulation (DBS) is an advanced treatment in Parkinson’s disease. We describe a 71-year-old patient in whom the DBS got infected with Mycobacterium bovis shortly after intravesical BCG instillations as an adjuvant treatment of bladder cancer. The DBS internal pulse generator and extension wires had to be replaced, and the patient was treated successfully with rifampicin, isoniazid, and ethambutol during three months. This case suggests that physicians need to be aware of the risk of this kind of infection and add a specific Mycobacterial test to the regular cultures.

## INTRODUCTION

Deep brain stimulation (DBS) is an established therapy for advanced Parkinson’s disease (PD), dystonia, tremor, some psychiatric disorders, and epilepsy. DBS leads are stereotactically implanted in the brain and connected through extension wires to an internal pulse generator (IPG), usually placed subcutaneously in the chest [1, 2]. DBS is a relatively safe surgical procedure, but complications, mainly infectious, are reported. We present the case of a PD patient with a Mycobacterium bovis infection of the DBS system following intravesical Bacillus Calmette-Guérin (BCG) instillation, as treatment for non-muscle-invasive bladder carcinoma (NMIBC). Local ethical board waived the need for a formal evaluation and the patient gave written informed consent.

## CASE

A 63-year-old man underwent bilateral DBS of the subthalamic nucleus (STN) for advanced PD. Although the clinical effect was excellent, 6 months later the IPG and extension wires, and later on also both leads, had to be explanted due to a *Staphylococcus Aureus* infection. He lacked medical or lifestyle factors influencing immunocompetence. The patient initially refused re-implant, but at the age of 67, underwent full bilateral DBS re-implant, again with a significant improvement. At age 70, he underwent a transurethral resection of a urothelial bladder tumor (TURBT) followed by adjuvant mitomycin treatment. Pathology showed high-grade urothelial cell carcinoma with infiltrative growth limited to the submucosa. Two months later, a second TURBT procedure with radical resection was performed, followed four weeks later by 15 subsequent adjuvant intravesical BCG injections during 9 months, according to the standard protocols. By the end of the treatment, he presented with a self-limiting wrist arthralgia, without signs of infection, possibly related to BCG treatment. A few weeks later he reported discomfort along the DBS extension wires. He had an erythematous rash around the IPG, suggestive for local infection. Laboratory tests demonstrated: leukocytes 7.3×10^9^/L (normal: 4.0–10.0×10^9^/L), C-reactive protein 11 mg/L (normal: <8 mg/L) and erythrocyte sedimentation rate 17/h (normal: <15 mm/h). He was afebrile and otherwise asymptomatic. IPG site fluid cultures were negative for common pathogens. Empiric antibiotic treatment with clindamycin 600 mg t.i.d. was started. Because of persisting local infection, 2.5 week later the IPG and both extension wires were replaced simultaneously, in order not to interrupt the stimulation. The old incisions were re-opened, the extension wires cut retro-auricular and a new path was tunneled to the contralateral infraclavicular site, where a new IPG was placed. Afterwards, the infected IPG was removed. Meanwhile, a mycobacterial culture of the aspirated fluid demonstrated the presence of Mycobacterium bovis. In the absence of other potential previous exposure to this pathogen, we diagnosed a disseminated Mycobacterium infection as complication of the intravesical BCG instillations. Chest X-ray ruled out pulmonary localizations. After consultation with a specialized pulmonologist, he was treated with rifampicin, isoniazid, and ethambutol for three months with complete recovery. Five months later, the urothelial cancer recurred, and was again successfully treated with TURBT and mitomycin, but without BCG instillation. Recovery persisted until the latest follow-up, five years after the DBS system re-implantation.

## DISCUSSION

We describe a case of DBS system infection with Mycobacterium bovis as a complication of BCG intravesical instillations.

Infections are the most common complications of DBS surgery, with a reported incidence rate of 0.62–14.3% depending on the definition applied [3, 4]. In almost half of the cases infections occur at the IPG site but can occur throughout the whole DBS system. The most common described pathogens are regular skin bacteria [4]. Late infections (>6 months after implantation) are often related to battery replacement, or skin erosions surrounding the hardware. Partial (33%) or complete (20%) removal of the DBS system can be indicated [3]. In our patient simultaneous re-implantation of new IPG and extension wires was performed, based on the symptoms severity and on the potential risk of DBS withdrawal syndrome. A recent case series reports successful simultaneous re-implantation, contralateral to the infected side, combined with antibiotic treatment in 5/6 patients [5].

BCG is a live, attenuated strain of Mycobacterium bovis. According to the European Association of Urology, adjuvant treatment with BCG has proven beneficial in preventing or delaying recurrence of intermediate/high risk NMIBC. The mechanism of the antitumor effect is unknown [6]. Side-effects of intravesical BCG treatment (hematuria (90%), cystitis (80%), fever (30,5%)) are common and usually seen in the first 48 h after instillation [7, 8]. Systemic osteoarticular side effects like arthralgia of one or multiple joints, as in our patient, are reported in 0.5–1% of the patients [9, 10]. Less than 5% of the patients have serious systemic complications [11]; these are generally referred to as ‘generalized BCGitis’ since Mycobacterium cannot always be isolated from the blood and an immune-allergic mechanism can be involved [8, 12]. Generalized BCGitis usually develops during intravesical BCG instillations; only in one immune compromised patient it was described years after the treatment [13]. It is associated with high fever and in 0.4% of the cases can lead to sepsis and multi organ failure, which can be lethal [11]. Laboratory tests can show an impaired hemodynamic status, leukopenia, and abnormal liver function. Also, BCG might symptomatically infect specific organs like lungs or liver. In case of a systemic BCG infection, treatment with isoniazid, rifampicin, and ethambutol during 6 months is described. Also high dose fluoroquinolones and corticosteroids are often administered, the last proven to prolong the survival [8, 11, 12, 14]. Generalized BCG-itis is mostly seen in the presence of (traumatic) lesions of the bladder during BCG instillations, leading to BCG entering the bloodstream [8]. Because of this risk, BCG is contra-indicated during the first 2 weeks after TURBT, after traumatic catheterization, and in patients with symptomatic urinary tract infection or visible hematuria. Immunocompromised condition is a relative contraindication [6]. Comorbidity burden seems to have no influence on the risk of developing systemic M. bovis infection after intravesical BCG [15]. Specific risk factors for implants infection cannot be derived from the current literature. Our patient had none of the risk factors for systemic spread of BCG and was immunocompetent.

No case of M. bovis infection of a DBS implant after BCG treatment has been reported so far. A case of a mycobacterial DBS infection (*Mycobacterium fortuitum*) at the IPG site has previously been described, unrelated to BCG treatment [16]. No infections were reported during a three-year follow-up period in a series of 143 patients with implants (artificial heart valves, pacemakers and orthopedic implants), receiving intravesical BCG instillations, suggesting that intravesical BCG instillations are relatively safe in patients with implants [17]. However, although rare, implants infections following intravesical therapy with BCG are described. The first case concerned an infected implantable cardiac defibrillator [18]. After this, multiple infections (*n* = 24) of implanted material have been reported, including prosthetic joint infections and vascular graft infections (Table 1). In the reported cases of infected vascular stents, it was not always clear whether the aneurysms were caused by the infection as well. Infection occurred between 2 months and 9 years after intravesical BCG instillations. All except one patient were males, aged 58–91 years (mean 75 years), reflecting the epidemiology of bladder cancer (average age of diagnosis 70–84 years) [19] and possibly increased general infection risk in the elderly. Dose reduction of BCG does not seem to reduce the number of severe events with respect to the full dose [9], while can increase the risk of recurrence of bladder cancer in higher risk tumors [8]. In the literature, the most frequently infected implants were orthopedic prostheses and vascular stents. Although theoretically a larger implant with bigger surface can represent a higher risk of infection, infections of smaller implants, including this DBS case, have been reported. Also, an intravascular graft could have a higher infection risk due to direct contact with M. bovis infected blood. However, the higher frequency of infection in these two kinds of implants can also depend on the higher prevalence of these implants. There are no guidelines for treatment of disseminated BCG infection, but in almost all cases the implanted material was explanted and patients were usually treated with isoniazid, rifampicin, ethambutol, and in some cases pyrazinamide. In literature, there is a variation in treatment duration ranging from six months to two years; only one case was treated during four months (ICD). The rationale for this variation is not quite clear but might depend on clinical recovery of the individual patients and/or negative culture [15, 20, 21, 22]. For the four patients in whom the implant was not removed, antibiotic treatment lasted at least 12 months. The choice of implant removal was sometimes based on persisting infection. Despite variation in management, all published cases recovered with no recurrence of M. bovis infection, even in the four patients in whom the implant was not explanted, suggesting that removal is not always necessary. Our case was treated successfully in only three months after replacement of the DBS, suggesting that a shorter duration of the treatment combined with replacement or removal of the implants might also be considered in selected cases, based on clinical recovery. However, with the current limited number of cases it is not possible to draw firm conclusions on the indications for treatment duration or removal.

**Table 1:** Published cases of implanted devices/prosthetics infected with M. bovis in the context of

	Case report	Sex and age (years)	Comorbidity	Number of instillations	Time between start/last instillation and infection, in months	Type of infected device/prosthetic	Mycobacterium in culture (site of taken culture)	Device removed	Treatment (duration)	Outcome (last follow up in months after start of therapy)
Electrical devices	
1	Our case	Male, 71	PD	15	9/1	DBS	Yes, M. bovis (IPG)	Yes, partly	Isoniazid, rifampin, ethambutol (3 months)	No signs of recurrent infection (48)
2	Stone, 1993 [18]	Male, 80	CABG, cardiac arrest	NR	5/NR	AICD	Yes, M. bovis (AICD)	Yes	Isoniazid, ethambutol (4 months)	No signs of recurrent infection (4)
Joint implants	
3	Chazerain, 1993 [24]	Male, 77	NR	NR	NR, diagnosis 2,5 months prior to infection	TKA	Yes, M. bovis	Two stage revision	Isoniazid, rifampin, ethambutol (2 year)	No signs of recurrent infection (24)
4	Guerra, 1998 [25]	Male, 66	Diabetes mellitus	12	14/11	THA	Yes, M. bovis (prothesis)	Yes, two stage revision	Isoniazid, rifampin (6 months)	Died 3 years later from unrelated cause
5	Segal, 2007 [22]	Male, 76	Osteoarthritis, aseptic loosening THA, spinal stenosis	NR	NR, diagnosis 4 years prior to infection	THA	Yes, M. bovis (prothesis, iliopsoas abscess)	Yes	Isoniazid, rifampin, ethambutol (1 year)	No signs of recurrent infection (36)
6	Reigstad, 2008 [26]	Male, 86	Osteoarthritis	9	2/0,5	THA	Yes, M. bovis (prothesis)	Yes	Isoniazid (2 year), rifampin (1 year), pyrazinamide (6 months)	No signs of recurrent infection (30)
7	Gomez, 2009 [27]	Male, 82	Osteoarthritis	6	25/28	THA	Yes, M. bovis (prothesis)	Yes	Isoniazid, rifampin (1 year)	No signs of recurrent infection (12)
8	Srivastava, 2011 [28]	Woman, 76	NR	17	NR, diagnosis 3 years prior to infection	THA	Yes, M. bovis (prothesis)	Yes	Tuberculostatics (6 months)	No signs of recurrent infection (5)
9	Aitchison, 2015 [29]	Male, 80	NR	6	9/7,5	THA	Yes, M. bovis (prothesis)	No, debridement and multiple washouts	Isoniazid, rifampin, ethambutol (15 months)	No recurrence of M. bovis (27), oral clindamycin for residual 3 mm sinus at hip with minimal discharge
10	Rispler, 2015 [30]	Male, 66	NR	NR	48/NR	TKA	Yes, M. bovis (prothesis)	No, incision and drainage	Isoniazid, rifampin (1 year)	No signs of recurrent infection (90)
11	Metayer 2018 [31]	Male, 70	Osteoarthritis, hepatocellular carcinoma	NR	29/28	THA	Yes, M. bovis (prothesis)	Yes	Isoniazid, rifampin, ethambutol (1 year), moxifloxacin (1 month, due to encephalopathy)	NR
12	Nguyen, 2019 [32]	Male, 90	Chronic kidney disease	12	48/NR	THA	Yes, M. bovis (prothesis)	No, incision and drainage	Isoniazid, rifampin, ethambutol (1 year)	No signs of recurrent infection (12)
13	Williams, 2019 [33]	Male, 70	Ankylosing spondylitis	12	78/67	THA	Yes, M. bovis (prothesis)	Yes	Ethambutol (3 months), moxifloxacin (9 months), isoniazid, rifampin (12 months)	No signs of recurrent infection (24)
14	Patel, 2019 [20]	Male, 91	Osteoarthritis post bilateral THA	NR	NR/36	THA	Yes, M. bovis (prothesis)	Yes	Isoniazid, rifampin (12 months), ethambutol (2 months)	No signs of recurrent infection (12)
15	Storandt, 2019 [34]	Male, 66	Osteoarthritis after pelvic fracture	6	24/ ‘few months’	THA	Yes, M. bovis (prothesis)	Yes	Isoniazid, rifampin, ethambutol (6 months)	No signs of recurrent infection (9)
16	Riste, 2021 [35]	Male, 79	Osteoarthritis of the spine, ischemic heart disease, benign prostatic hyperplasia, pulmonary embolism	6	3/<1	THA	Yes, M. bovis (prothesis)	Yes	Isoniazid, rifampicin, isoniazid, ethambutol and moxifloxacin (6 months)	No signs of recurrent infection
17	Seelig, 1999 [36]	Male, 72	Peripheral arterial occlusive disease	NR	NR, diagnosis 6 months prior to infection	Femofemoral bypass (polytetrafluorethylene graft) and bilateral femoropopliteal bypass (reverse saphenous vein)	Yes, M. bovis (graft)	Yes	Isoniazid, ethambutol, rifampin (NR)	No signs of recurrent infection (3)
18	Shakir, 2009 [37]	Male, 62	Peripheral artery disease	15	10/^1^/_4_	Axillary-femoral bypass graft	Yes, M. bovis	Yes	Isoniazid, rifampin, ethambutol (9 months)	NR
19	Santbergen 2013* [38]	Male, 58	Discopathy L4-L5, MI, stroke, prostatectomy, EVAR AAA	NR	36	EVAR	Yes, M. bovis (psoas abscess)	Yes	Isoniazid, rifampicin, pyrazinamide, ethambutol (1 year)	No signs of recurrent infection (30)
20	Mizoguchi, 2013^∧^ [39]	Male, 81	AAA, epidimytis M bovis (48 months after BCG)	NR	54 months	Stent graft AAA	Yes, M. bovis (iliopsoas, fistula to AAA)	Yes	Rifampicin, isoniazid (NR)	NR
21	DeSimone, 2016 [40]	Male, 82	Aortic atherosclerosis	NR	72/4	Axillo-bifemoral femoral bypass graft	Yes, M. bovis (graft) and S. aureus	Yes	Isionazid, firampin (9 months)	No signs of infection (13)
22	Meyer, 2020 [21]	Male, 85	COPD, CAG, hypertension, spinal stenosis, treatment pembrolizumab	NR	NR, start therapy 12 months prior to presentation	Femofemoral bypass (polytetrafluorethylene graft)	Yes, M. bovis (graft)	Yes	Isoniazid, rifampin (6 months)	No signs of recurrent infection (3)
23	Buerger, 2021 (case A)* [41]	Male, 63	COPD, Disc prolapse L5-L6, renal cysts, ruptured AA	NR	NR (start therapy 4 years prior to presentation)	Aortobi-iliac graft NB placed 2 years after instillations	Yes, M. bovis (retroperitoneal mass, growth to graft)	No	Isoniazid, rifampin (12 months), ethambutol (6 months)	No signs of recurrent infection (84)
24	Buerger, 2021 (case C) [41]	Male, 79	CAD, hypertension, hyperlipidemia, cardiac arrhythmia type II Wenkebach	36	NR (start therapy 9 years prior to presentation)	Dacron tube graft aneurysm NB placed 7 years after instillations	Yes, M. bovis (graft)	Yes	Isoniazid, ethambutol, rifabutin instead of rifampicin because drug interaction (NR)	No signs of recurrent infection (12)
25	Dubert 2021 [42]	Male, 70	Aortobifemoral bypass, arteriopathy, ventral hernia abdomen	18	24/10	Aortobifemoral bypass and abdominal wall	Yes, M. bovis, also S. intermedius	Yes	Isoniazid, rifampicin, ethambutol (9 months) Pyrazinamide (temporary) Antibiotics	Recurrence of S. intermedius after 3 months. No recurrence of M. bovis (28)
26	Arsuffi, 2023 [43]	Male, 73	Hypertension, diabetes, chronic kidney disease, ischemic cardiomyopathy prostatic adenoma	NR	NR/2	Femoral-popliteal bypass	Yes, M. bovis	Yes	Rifampin, ethambutol, isoniazid (11)	No signs of recurrence

^*^Aneurysm due to M. bovis infection not excluded, ^∧^Spread of infection to graft from infection site elsewhere. NR, not reported; AAA, abdominal aortic aneurysm; PD, Parkinson’s disease; DBS, deep brain stimulator; IPG, internal pulse generator; CABG, coronary artery bypass graft; AICD, automatic implantable cardiac defibrillator; COPD, chronic obstructive pulmonary disease; CAD, coronary artery disease; TKA, total knee arthroplasty; THA, total hip, arthroplasty.

### Conclusion

Intravesical BCG instillation is a standard treatment in cases of NMIBC, a frequent tumor in elderly patients [23]. Although rare, the systemic spread of Mycobacterium bovis can form a potentially serious risk in patients with implanted devices including DBS. Although BCG treatment is reported to be generally safe, also in patients with different kinds of prosthetic devices, patients and treating physicians should be aware of the infection risk, and standard measures should be taken to avoid systemic spread. Also, physicians should actively add mycobacterial culture to the standard cultures in case of infection in patients who underwent BCG treatment. Based on the current literature, it is not possible to give a general advice about a suitable management, which should rely on patient specific considerations.
